# The beginning of a new therapeutic era in acute myeloid leukemia

**DOI:** 10.1002/jha2.252

**Published:** 2021-07-27

**Authors:** Christian Récher

**Affiliations:** ^1^ Service d'Hématologie Centre Hospitalier Universitaire de Toulouse Institut Universitaire du Cancer de Toulouse Oncopole Université Toulouse III Paul Sabatier Centre de Recherches en Cancérologie de Toulouse Toulouse France

**Keywords:** acute myeloid leukemia, CPX‐351, enasidenib, FLT3 inhibitors, gemtuzumab ozogamycin, gilteritinib, glasdegib, IDH inhibitors, ivosidenib, midostaurin, monoclonal antibodies, oral azacitidine, TP53, venetoclax

## Abstract

In the field of AML, the early 2000s were shaped by the advent of novel molecular biology technologies including high‐throughput sequencing that improved prognostic classification, response evaluation through the quantification of minimal residual disease, and the launch of research on targeted therapies. Our knowledge of leukemogenesis, AML genetic diversity, gene‐gene interactions, clonal evolution, and treatment response assessment has also greatly improved. New classifications based on chromosomal abnormalities and gene mutations are now integrated on a routine basis. These considerable efforts contributed to the discovery and development of promising drugs which specifically target gene mutations, apoptotic pathways and cell surface antigens as well as reformulate classical cytotoxic agents. In less than 2 years, nine novels drugs have been approved for the treatment of AML patients, and many others are being intensively investigated, in particular immune therapies. There are now numerous clinical research opportunities offered to clinicians, thanks to these new treatment options. We are only at the start of a new era which should see major disruptions in the way we understand, treat, and monitor patients with AML.

## 1 INTRODUCTION

It has long been written that there was no breakthrough for the treatment of acute myeloid leukemia (AML) as compared to other hematological malignancies including chronic myeloid leukemia, B‐cell lymphoma (BCL), or multiple myeloma [1]. Going back more than 20 years ago, the situation was roughly similar for multiple myeloma. Younger patients were treated with high‐dose melphalan and autologous stem‐cell transplantation while older patients received low‐dose melphalan and prednisone, the so‐called MP regimen that has fallen into oblivion over the years [2]. Now, there have been more than 10 novel drugs approved in myeloma targeting intracellular pathways, tumor microenvironment, and cell surface antigens the combinations of which have transformed a highly deadly disease into a chronic one [3, 4]. In AML, we have been relying for the past 40 years on the combination of cytarabine and an anthracycline, so‐called (“7+3″), as induction chemotherapy in patients suitable for intensive treatments followed by high‐dose cytarabine consolidation and eventually by allogeneic stem cell transplantation (cure being the goal), whereas older or unfit patients received low‐dose cytarabine or more recently hypomethylating agents, both inducing few complete responses (CRs) and little hope for cure (prolonged survival being the goal) [5]. Then, genomics changed the game. Firstly, targeted gene sequencing successively identified *FLT3*‐ITD, *CEBPA*, and *NPM1* mutations which helped improve prognostic classification, the response evaluation through quantification of molecular residual disease, and launched the research on targeted therapies leading to the registration of midostaurin as the first FLT3 inhibitor in AML [6, 7]. Soon after, thanks to the advance in high‐throughput sequencing technologies, the first AML genome was reported in 2008 and subsequent studies identified several novel recurrent mutations with pathophysiological, prognostic, or therapeutic relevance such as *IDH1*, *IDH2*, or *TP53* mutations [8, 9, 10]. Our knowledge of leukemogenesis, AML genetic diversity, gene‐gene interactions, clonal evolution, and treatment response assessment has also greatly improved, and new classifications based on chromosomal abnormalities and gene mutations are now integrated in clinical environments [11, 12, 13, 14, 15, 16, 17]. These considerable efforts contributed to the discovery and development of promising drugs for AML specifically targeting gene mutations, apoptotic pathways, and cell surface antigens. Novel liposomal formulations of classical cytotoxic agents are also promising. Midostaurin, gemtuzumab ozogamycin, glasdegib, venetoclax, ivosidenib, enasidenib, gilteritinib, CPX‐351, and oral azacitidine were approved by the Food and Drug Administration (FDA) of for AML patients in less than 3 years between 2017 and 2020 [18, 19] (Table 1).

**TABLE 1:** Novel drugs recently approved in acute myeloid leukemia

Agent	Approval	Mechanism of action	Indication
Midostaurin	2017	FLT3 inhibition	Newly diagnosed AML with *FLT3* mutation in combination with standard induction, consolidation +/‐maintenance
Enasidenib	2017	IDH2 mutant inhibition	Relapsed or refractory AML with *IDH2* mutation
CPX‐351	2017	Liposomal formulation including daunorubicin and cytarabine at a fixed 5‐molar:1‐molar ratio	Newly diagnosed, therapy‐related AML (t‐AML) or AML with myelodysplasia‐related
changes (AML‐MRC)			
Gemtuzumab ozogamicin	2017	CD33 monoclonal antibody linked to calicheamicin	Newly diagnosed, CD33‐positive AML in combination with standard induction and relapsed or refractory CD33‐positive AML
Ivosidenib	2018	IDH1 mutant inhibition	Relapsed or refractory or newly diagnosed (unfit) AML with *IDH1* mutation
Glasdegib	2018	Hedgehog pathway inhibition	Unfit or older (≥75y) patients with newly diagnosed AML in combination with low‐dose cytarabine
Venetoclax	2018	Bcl‐2 inhibition	Unfit or older (≥75y) patients with newly diagnosed AML in combination with hypomethylating agents or low‐dose cytarabine
Gilteritinib	2018	FLT3 inhibition	Relapsed or refractory AML with *FLT3* mutation
Oral azacitidine	2020	Hypomethylating agent	Continued treatment of AML patients who achieved CR/CRi following intensive induction chemotherapy and are not able to complete intensive curative therapy (i.e., alloSCT)

Rather than just another review stacking up one novel drug after another, this article will try to outline the perspectives for the coming years in the field of AML [20, 21]. Indeed, there are now numerous clinical research opportunities offered to clinicians with these new molecules and with those under development. With this sudden over‐abundance of choices, we feel like a lottery player who gets rich overnight and wonders what he's going to do with it all!

## 2 VENETOCLAX IN AML, THE MAGIC POTENTIATOR

The anti‐apoptotic BCL 2 (BCL‐2) protein is overexpressed in AML, especially in leukemic stem cells that are supposed to be responsible for chemoresistance and relapse. BCL‐2 overexpression is a poor risk factor in AML and is associated with chemoresistance [22]. BCL‐2 inhibition by small molecule inhibitors kills AML blasts, targets oxidative phosphorylation, and selectively eradicates leukemic stem cells [23, 24]. In AML patients, contrary to chronic lymphocytic leukemia, venetoclax – an oral, selective, small‐molecule inhibitor of BCL‐2 – is not very active as a single agent with perhaps, the exception of *NPM1* or *IDH* mutation subgroups [25, 26]. However, when combined to low‐dose cytarabine or with hypomethylating agents, venetoclax dramatically increases the CR rates, thus demonstrating synergistic activity in patients. Venetoclax has been recently approved in combination with hypomethylating agents as first line therapy in AML patients who are ineligible to receive standard induction therapy on the basis of high response rates and promising response durations in a Phase 1b/2 trial [27, 28]. These results have been very recently confirmed in the randomized, placebo‐controlled, VIALE‐A Phase 3 trial which demonstrated the superiority of azacitidine plus venetoclax over azacitidine plus placebo in terms of CR rate (66.4% vs. 28.3%), duration of response (17.5 vs. 13.4 months), and OS (14.7 vs. 9.6 months) [29]. These differences are highly statistically and clinically significant. This major breakthrough is reminiscent of the one made more than 40 years ago when anthracyclines were added to cytarabine in patients eligible for intensive chemotherapy. Furthermore, the CR rate with azacitidine‐venetoclax is >70% in the subgroups with *IDH1* or *IDH2* mutations [30]. These unprecedented results compared favorably with intensive chemotherapy in fit patients and generate hope for a cure in some patients. With the exception of the risk of tumor lysis syndrome and increased incidence of febrile neutropenia, no unexpected adverse event emerged, thus ensuring the widespread use of this combination in this difficult‐to‐treat population of unfit or elderly patients. Venetoclax combined with low‐dose cytarabine was also superior to low‐dose cytarabine in the VIALE‐C Phase 3 trial [31].

Thus, doublet azacitidine‐venetoclax is becoming the new standard of care in patients ineligible for intensive induction chemotherapy and the standard arm to overcome in clinical trials. Planned or on‐going clinical trials are already comparing this doublet to triplets with small molecule inhibitors or monoclonal antibodies. For example, the first results of the azacitidine‐venetoclax‐ivosidenib triplet may reach a 100% rate of CR in treatment‐naïve AML patients with *IDH1* mutations (EHA Library. DiNardo C. 06/12/20; 294963; S143).

There is an on‐going debate on how to define patients unfit for intensive induction chemotherapy [32, 33, 34]. There are no absolute clear‐cut criteria to unambiguously select the patients, and certain parameters used in the majority studies, such as performance status (a highly subjective criterion) or age, are questionable in this setting [34]. It has been suggesting that comorbidity, physical capacities, and nutritional status may be more relevant than performance status or age [35]. What about a 70‐year‐old patient with a normal karyotype, an ECOG performance status of 2 and no comorbidity? In routine, this patient is fit for chemotherapy especially when no valuable alternative is available [36]. However, patients of the VIALE‐A study could be included in the trial on the sole basis of the ECOG criteria of 2; therefore it is likely that this Phase 3 trial has included a substantial number of patients that were otherwise fit for chemotherapy outside clinical trials. Therefore, many physicians will be tempted to replace intensive chemotherapy by azacitidine‐venetoclax in older patients since CR rate and survival are comparable, and toxicity is likely to be lower with the doublet, although this important question should be the matter of prospective randomized trials.

Beyond the population of unfit patients, venetoclax is likely to become the drug of choice for combination therapy in virtually all AML patients. In relapse or refractory AML, the preliminary results of combinations with other small molecules inhibitors such as FLT3, IDH, or MDM2 inhibitors seem very promising [37, 38]. In patients fit for intensive chemotherapy, adding venetoclax to 7+3 is feasible and induces promising response rates, especially in patients with intermediate cytogenetic risk or with *NPM1* or *IDH* mutations [25]. In these fit patients who have achieved CR after intensive induction chemotherapy, venetoclax in combination with intermediate dose cytarabine as consolidation therapy and/or with azacitidine as maintenance therapy so as to eradicate residual disease will be an important scope of investigation. To date, 91 clinical trials with venetoclax in AML are recorded on the clinicaltrials.gov Website, thus highlighting the tremendous interest for this drug in AML. This has been confirmed at the virtual 2020 American Society of Hematology meeting where no less than 10 oral communications dealt with venetoclax based‐combinations. Venetoclax is becoming the drug of choice to combine with all kind of therapeutic strategies in AML including high or low intensity chemotherapy, novel agents, and tyrosine kinase inhibitors. In a phase 2 trial, a low‐intensity backbone of cladribine/low dose cytarabine plus venetoclax alternating with azacitidine plus venetoclax for older patients with AML, yielded very high rates of durable MRD negative CR (96% CR/CRi and 80% MRD negative). With a median follow‐up of 11+ months, the median OS was not reached, with 6‐ and 12‐month OS rates of 86% and 70%, respectively [39]. Venetoclax has been also combined with intensive chemotherapy including CPX‐351 or FLAG‐Ida regimen [40, 41]. These combinations are feasible provided that the dose and duration of venetoclax exposure is reduced. Prolonged myelosuppression and infections remain a major problem with this type of combinations which will be reserved for suitable patients. In FLT3 mutated AML, combining venetoclax plus FLT3 inhibitors such as quizartinib or gilteritinib with or without hypomethylating agents also induced impressive response rates in R/R AML patients [42, 43]. However, AML patients with *TP53* mutations did not appear to benefit from venetoclax based‐treatment. Indeed, the combination of venetoclax and decitabine was associated with inferior response rate, shorter response duration, higher MRD positivity, and a poor median OS of 5.2 months [44].

## 3 IDH AND FLT3 INHIBITORS, THE SMART DRUGS WITH COMPANION BIOMARKERS

With the development and recent approval of drugs targeting specific mutations such as FLT3, IDH1, and IDH2, it has become critical that onco‐hematology laboratories increase their turnaround in order to allow clinicians to prescribe these agents in a timely manner. It has long been postulated that AML represents an oncologic emergency and should be treated without delay, especially in younger patients treated by intensive chemotherapy [45]. However, recent studies have proven that waiting a short period of time so as to characterise molecular alterations and design tailored treatments at diagnosis is safe [46, 47]. It is now crucial that clinicians be knowledgeable on a panel of gene mutations including at least *FLT3*, *IDH1*, *IDH2*, *NPM1*, *CEBPA*, and *TP53* at the time of diagnosis (48–72 h) in order to guide initial treatment. Furthermore, since AML clones and subclones are subject to clonal evolution under the therapeutic pressure of either chemotherapy or targeted agents, repeating molecular testing in each phase of the disease (refractory or relapse) should be mandatory. Recent advances in the treatment of AML with FLT3 or IDH inhibitors illustrate well this recent development.

### 3.1 IDH1 inhibitors

Somatic mutations of isocitrate dehydrogenase 1 (*IDH1* ^R132^) genes are found in 6%–10% of AML patients [48]. *IDH1* mutations are most frequent in AML with normal karyotype and associated with *NPM1* and DNMT3A mutations at diagnosis [49]. Their prognostic impact mainly depends on the mutational context [50, 51, 52]. Furthermore, *IDH1* mutations, which have been described in clonal hematopoiesis, are considered as early event during leukemogenesis, stable at relapse and thus, have emerged as promising therapeutic targets. It is also noteworthy that the molecular landscape of AML with *IDH1* mutations observed at R/R disease under chemotherapy selection pressure differs from diagnosis with an increased frequency of *SRSF2*, *ASXL1*, *RUNX1*, *NRAS*, and *TP53* co‐occurring mutations [49, 53].

Ivosidenib – an oral, targeted, small‐molecule inhibitor of mutant IDH1 – has been evaluated as a single agent in a Phase 1 study in relapsed or refractory (R/R) AML with *IDH1* mutation [53]. The frequency of Grade 3 or higher treatment‐related adverse events was low, mainly a prolongation of the QT interval, leukocytosis, and differentiation syndrome which are manageable [54]. CR or CR with partial hematologic recovery (CRh) was 30.4% with 21.8% CR, whereas CRi was 11.7%. It should be noted that clonal or subclonal m*IDH1* had similar CR/CRh rates. Furthermore, mutation clearance was observed in 21% of responding patients, thus demonstrating that deep response may be achieved in some patients. The median duration of response was 9.3 months in CR patients. The median OS was 8.8 months. Based on these promising results, ivosidenib was recently approved by the FDA.

The mechanisms of resistance to ivosidenib were studied in patients who failed to or relapsed after the response to this drug [55]. Receptor tyrosine kinase pathway mutations and mutations in *NRAS* and *PTPN11* were significantly associated with the lack of response to ivosidenib. Interestingly, emerging mutations in patients who relapsed or progressed under ivosidenib were *IDH* or non‐*IDH*‐related. Indeed, mutations of resistance in a second site of *IDH1* or the emergence of *IDH2* ^R140^ clones were detected in ∼25% of resistant patients, whereas potentially actionable mutations in genes such as *FLT3*, *NRAS*, or *KRAS* were also identified, thus indicating that molecular rescreening is important at each stage of the disease.

The preliminary results of ivosidenib combined with azacitidine when treating naive patients showed a CR rate of 70% and may reduce the emergence of mutant resistant clones [56].

### 3.2 IDH2 inhibitors

Somatic mutations of the *IDH2* gene, either *IDH2* ^R140^ or *IDH2* ^R172^, occur in 5%–15% and 1%–4% of AML, respectively [48]. Like IDH1, *IDH2* mutations are frequently found in normal karyotype AML [57, 58]. At diagnosis, *IDH2* ^R140^ mutations are associated with *NPM1* and *DNMT3A* mutations whereas in the relapse/refractory setting, mutations of the *SRSF2*, *DNMT3A*, *RUNX1*, *ASXL1, NRAS*, and *BCOR* genes emerge as the most frequent co‐mutations [11, 48, 59]. *IDH2* ^R172^ mutations are associated with *DNMT3A* and *BCOR* mutations and mutually exclusive with *NPM1* and other class‐defining mutations [49]. Therefore, AML with *IDH2* ^R172^ has been recognized as a defined subgroup of the AML genomic classification [11].

Enasidenib – an oral, targeted, small‐molecule inhibitor of mutant IDH2 – has been evaluated as a single agent in a Phase 1 study in mutant *IDH2* R/R AML patients and subsequently approved by the FDA [60]. A low frequency of Grade 3 or higher treatment‐related adverse events was reported, mainly leukocytosis and differentiation syndrome [59, 61]. The overall response rate was 40.3% including 19.3% CR and 6.8% CRi. The median OS was 9.3 months and reached 19.7 months in CR patients. CRs were observed in patients with subclonal IDH2 mutations, and a variant allele frequency of IDH2 mutant, which measures the mutational burden, was not associated with the response. Also, whereas in some CR patients, *IDH2* mutation clearance was achieved, *IDH2* mutational burden did not decrease in all responding patients during treatment, possibly due to the maturation of leukemic blasts into the functional neutrophils carrying the mutation. The mechanisms of resistance may involve the emergence of second‐site IDH2 mutations, IDH2‐mutant subclones with neomorphic mutations in *IDH1*, co‐occurring mutations in NRAS, and other MAPK pathway effectors or complex clonal evolution [59, 62, 63].

A recent randomized Phase 2 trial of azacitidine versus azacitidine plus enasidenib in newly diagnosed AML patients unfit for intensive chemotherapy showed a significantly higher CR rate with the combination compared to azacitidine alone (53% vs. 12%) and a median duration of response of 24.1 months in the combination arm (EHA Library. DiNardo C. 06/12/20; 294959; S139).

Whereas IDH inhibitors in combination with azacitidine yielded some very interesting response rates compared to azacitidine alone, one open issue will be to determine which induction regimen to choose between azacitidine‐venetoclax and azacitidine‐IDH inhibitors. Will it be more appropriate to start with the standard azacitidine‐venetoclax and reserve the IDH inhibitors for relapse? Or will triplets eradicate the disease at first line? Which inhibitor will we use in patients with other concomitant targetable mutations, such as FLT3 or TP53? In fit patients, the addition of ivosidenib or enasidenib to intensive chemotherapy is under evaluation in an international Phase 3 trial involving several cooperative AML study groups (NCT03839771). Several others IDH1 or IDH2 inhibitors including drugs that target both mutations are under investigation [64].

### 3.3 FLT3 inhibitors

Mutations in the *FLT3* gene are among the most common mutations in AML, occurring in up to 30% of patients [65]. There are two distinct activating *FLT3* mutations: internal tandem duplications (ITD) in the juxta‐membrane domain and point mutations in the tyrosine kinase domain 2 (TKD). *FLT3* mutations are associated with an aggressive disease course, especially *FLT3*‐ITD which generally predicts an early relapse and poor prognosis. Through clonal selection under chemotherapy, a higher mutant allelic burden is frequently observed at relapse, thus indicating that AML cells have become more addicted to FLT3 signalling. This is an important point because at least in preclinical settings, FLT3‐mutant allelic burden and clinical status (i.e., diagnosis versus relapse samples) are predictive of the response to FLT3 inhibitors in AML [66].

Midostaurin – a staurosporine analog with a multikinase inhibitory activity against KIT, PDGFR, PKC, VEGF, and FLT3 (amongst others) – was the first FLT3 inhibitor to be approved for frontline therapy in AML patients with FLT3 mutations and fit for intensive chemotherapy [67]. In the randomized Phase 3 trial RATIFY, midostaurin and placebo were added to standard “7+3″ induction chemotherapy and high‐dose cytarabine consolidation followed by a 12‐months single agent maintenance in younger patients (18–60 years) with *FLT3*‐ITD or *FLT3*‐TKD mutations. The CR rates were similar in both groups. However, midostaurin significantly improved the 4‐year overall OS from 44.3% to 51.4%, compared with placebo [7]. The benefit of midostaurin was observed in *FLT3*‐ITD patients, whatever the allelic ratio, and in *FLT3*‐TKD patients. A subsequent exploratory analysis based on the *FLT3*‐ITD patient of the RATIFY trial showed that the impact of midostaurin was significant in the three prognostic subgroups of the ELN2017 classification which includes NPM1, RUNX1, ASXL1, and TP53 as well as cytogenetic risk [68]. Furthermore, the benefit of midostaurin was independent from the allogeneic stem cell transplantation. A Phase 2 study suggested that older patients aged 60–70 years may also benefit from midostaurin [69]. Midostaurin was approved in Europe for induction, consolidation, and maintenance, whereas it was only approved for induction and consolidation in the US indicating that the need for midostaurin as maintenance is uncertain. However, this postulate could also apply to the consolidation phase since RATIFY was not designed to demonstrate that midostaurin treatment beyond induction chemotherapy was essential. Anyhow, since this approval and because FLT3 mutated patients present with a high tumor burden at diagnosis, the results of FLT3 mutational screen must been found rapidly.

Two randomized Phase 3 trials with second generation FLT3 inhibitors were recently conducted in R/R AML patients with *FLT3* mutations (gilteritinib) or *FLT3*‐ITD only mutations (quizartinib) [70, 71]. In both studies, the FLT3 inhibitor as a single agent was superior to the standard of care with high or low intensity chemotherapy in terms of response and OS. However, while gilteritinib has been broadly approved in North America, Europe and Japan, quizartinib was only registered in Japan.

Gilteritinib is an oral, small molecule inhibitor, highly selective of FLT3 acting against both *FLT3*‐ITD and *FLT3*‐TKD mutations and only acting marginally against cKIT [72, 73] which distinguishes it from quizartinib and likely explains the weak myelosuppression observed in patients. Gilteritinib also targets AXL, another tyrosine kinase implicated in the resistance to chemotherapy and FLT3 inhibitors [74, 75]. In the pivotal Phase 3 ADMIRAL study, AML patients with R/R FLT3‐mutated AML were randomized between 120‐mg/day gilteritinib and a standard of care with high or low intensity regimen defined by physicians prior to randomization [70]. It is important to keep in mind that few patients of the ADMIRAL trial had previously been exposed to midostaurin, which is no longer the case. Gilteritinib induced higher CR/CRh and CR rates (34.0% vs. 15.3% and 21.1% vs. 10.5%, respectively) and significantly improved OS (median OS, 9.3 vs. 5.6 months). Adverse events were more frequent in the standard chemotherapy arm, with the exception of liver transaminase elevations. QTc prolongation, differentiation syndrome, and lipase elevation are very rare events in the context of gilteritinib treatment (<5%), whereas posterior reversible encephalopathy syndromes have been exceptionally reported [54, 76]. Off‐target activating mutations in genes of the *RAS/MAPK* pathway have been identified as a key mechanism of the resistance to gilteritinib and confirmed in patients of the ADMIRAL trial who relapsed on gilteritinib treatment in whom in‐target *FLT3*‐F691L mutations were also detected [77, 78].

Phase 3 randomized trials comparing first line intensive chemotherapy plus midostaurin or second generation inhibitors are ongoing, and many combinations with hypomethylating agents or targeted agents are also being investigated [79]. Furthermore, other novel FLT3 inhibitors are under development, and it is foreseeable that clinicians may have a handful of FLT3 inhibitors in order to deal with clonal evolution, drug‐drug interactions, adverse events, or co‐morbid conditions just like BCR‐ABL inhibitors for Philadelphia‐chromosome‐positive leukemia [80, 81, 82, 83].

Patients with *FLT3*‐ITD mutations are generally candidates to allogeneic stem cell transplantation, even though the post‐transplant relapse rate remains a problem [84, 85]. Interestingly, sorafenib – a multikinase inhibitor with potent activity against FLT3 – has demonstrated clinical activity in *FLT3*‐ITD patients having relapsed after transplantation [86]. A subsequent comprehensive preclinical study elegantly demonstrated that sorafenib, like other FLT3 inhibitors, increased the IL‐15 production by *FLT3*‐ITD leukemic cells leading to the potentiation of the allogeneic CD8+ T cell response as well as disease eradication in preclinical models [87]. As a clinical translation of this finding, the randomized Phase 3 SORMAIN trial demonstrated that sorafenib maintenance therapy reduces the risk of relapse and death after transplantation in AML patients with *FLT3‐*ITD mutations [88]. Reducing the risk of relapse after allogeneic stem cell transplantation by maintenance therapy with non‐cytotoxic drugs is an active field of research going beyond FLT3 inhibitors, and virtually all the small molecules inhibitors having shown efficacy in AML will be assessed in this context [89, 90].

## 4 TP53: WANTED, DEAD, OR ALIVE

Although the *TP53* tumor suppressor gene is the most frequently mutated gene in human cancer, its incidence in AML is relatively low (5%–20%) and increases with age or in therapy‐related AML [48]. Patients with *TP53* mutations have one of the worst prognosis in AML because their disease is both chemo and immune resistant, as shown by the poor response rate to standard treatments including intensive chemotherapy and hypomethylating agents and the high rate of relapse after allogeneic stem cell transplantation [91, 92, 93, 94]. Treatment of this AML subgroup is an urgent unmet medical need, and therefore, huge efforts have been made to drug the undruggable. The first glimmer of hope came from eprenetapopt (APR‐246), which is the first‐in‐class small molecule that selectively targets *TP53* mutated cancer cells [95, 96]. Furthermore, eprenetapopt acts in synergy with azacitidine [97]. In two recent phase 2 trials designed for *TP53* mutated myelodysplastic syndrome or AML, eprenetapopt combined with azacitidine induced an overall response rate of 62%–71% (44%–47% CR) with a median duration of response at 8–10.4 months. [98, 99]. Neurologic toxicity emerged as the main adverse effect with this drug. Furthermore, it has been recently shown that AML with *TP53* mutations are associated with an infiltration of cytotoxic lymphocytes in the tumor microenvironment, indicating that immune intervention could be of value in this subgroup of patients [100, 101]. Altogether and even though these data are immature, there is reasonable hope to improve the outcome of patients with TP53 mutated‐AML in the near future.

## 5 CHEMOTHERAPY'S NOT DEAD (YET)

A sizable proportion of intermediate and good‐risk AML patients are cured by intensive chemotherapy in one shot (i.e., without the need for allogeneic stem cell transplantation and without relapse). For these patients who generally receive one 7 + 3 induction cycle then three cycles of intermediate‐to‐high dose cytarabine, treatment is definitely completed in 6 months, and thereafter, the patients are treatment‐free for the rest of their life. In these patients, the added value of new drugs will be challenging to demonstrate both in terms of efficacy and duration of treatment because most new drugs have been developed so as to be administered on a long‐term basis. However, well‐known adverse events including profound myelosuppression, gastro‐intestinal toxicity, severe mucositis, and infections as well as the strong impact of chemotherapy on quality of life remain of concern. CPX‐351, a dual‐drug liposomal combination of daunorubicin and cytarabine with a synergistic drug ratio, was approved for the treatment of adults with newly diagnosed therapy‐related AML or AML with myelodysplasia‐related changes [102]. Long‐term results of the pivotal phase 3 trial shown at ASH 2020 confirmed the superiority of CPX‐351 over standard “3+7″ chemotherapy and the particular good outcome of patients who were allografted in first response after CPX‐351 [103]. Interestingly, CPX‐351 accumulates in the bone marrow where it has been shown to be taken up to a greater extent by AML cells than normal bone marrow cells and sparing normal tissues [104]. In a clinical context, this translates into improved efficacy (a higher response rate) but also into increased tolerability to induction chemotherapy. Nurses were probably among the first to notice this curious effect of CPX‐351 compared to free daunorubicin and cytarabine: much less mucositis, gastrointestinal (GI) toxicity, and no hair loss. It is generally recognized by caregivers that CPX‐351 is better tolerated than the standard 7 + 3, even though the Phase 3 trial did not clearly demonstrate this point. Furthermore, when ranking adverse events, it is noteworthy that hair‐loss is the most common side effect reported as severe by the patients, while caregivers are more prone to declare infections, suggesting that quality of life with CPX‐351 should be better than with 7+3 [105]. Therefore, it is tempting to foresee that CPX‐351 indications could extend beyond the label to be broadly applied in *de novo* AML. In this regard, the results of the ongoing Phase 3 trial of the German AMLSG study group, currently assessing CPX‐351 versus standard “7+3″ in newly diagnosed AML and intermediate‐ or adverse genetics (≥18 y) (NCT03897127), will be of great importance for AML patients fit for chemotherapy.

## 6 REVISITING THE CONCEPT OF REMISSION MAINTENANCE WITH ORAL THERAPY IN AML

Preventing relapse after induction and consolidation therapy in AML has long been a major challenge especially in older patients ineligible for allogeneic stem cell transplantation [106]. While there have been some interesting data with drugs such as the histamine dihydrochloride‐interleukin 2 combination, which was associated with prolonged disease‐free survival or more recently with norethandrolone which improved OS, there have been no approved drug in this indication [107, 108]. CC‐486, an oral formulation of azacitidine has been recently developed for maintenance therapy in AML. Oral dosing of this drug that is not bioequivalent to standard injectable azacitidine, allows for extended drug exposure and prolonged pharmacodynamic effects. CC‐486 was recently approved for maintenance of first CR after intensive chemotherapy in adult patients with AML not able to proceed to allogeneic stem cell transplantation. Indeed, in the pivotal randomized phase 3 trial (QUAZAR AML‐001) that randomized 472 patients who were 55 years or older, in first CR after intensive chemotherapy and not candidates for transplantation, oral azacitidine improved OS compared to placebo (median OS, 24.7 vs. 14.8 months) [109]. Several communications at 2020 American Society of Hematology provided a better understanding of the QUAZAR AML‐001 results. Indeed, the comparison of patients who received no consolidation (20% of the cohort), 1 consolidation (45%), or ≥2 consolidations (35%), showed that CC‐486 was associated with consistent survival benefits vs. placebo regardless of number of prior consolidation cycles ([110]). Furthermore, this study included long‐term longitudinal assessment of MRD. CC‐486 improved OS compared to placebo in patients who were either MRD+ (median 14.6 vs. 10.4 months) or MRD‐ (median 30.1 vs. 24.3 months) at study entry and induced in a higher rate of MRD+ to MRD‐ conversion (37% vs. 19%) [111]. GI events were the most common treatment‐emergent adverse events reported in patients who received CC‐486. These GI events were low‐grade and decreased in frequency after initial treatment cycles [112]. Beyond the maintenance phase, there is much to be anticipated with this oral hypomethylating agent that could replace the injectable form in other phases of treatment alone or in combination with new agents.

## 7 MONOCLONAL ANTIBODIES: WILL THE SECOND WAVE HAPPEN?

B‐cell non‐Hodgkin lymphoma has rituximab; multiple myeloma has daratumumab and acute lymphoblastic leukemia blinatumomab. What about AML? After small molecules inhibitors, the second wave of new game‐changing drugs in AML may be represented by monoclonal antibodies. There is indeed a great deal of excitement in AML, thanks to various immune therapies, including naked antibodies targeting surface antigens expressed by leukemic stem cells, bispecific T‐cell engager antibodies, or antibodies targeting immune checkpoint receptors [113, 114]. Naked monoclonal antibodies targeting CD47, CD70, or TIM3 that are expressed on leukemic stem cells display a very good safety profile as single agent and are excellent candidates for combination with hypomethylating agents. For example, anti‐CD47 magrolimab combined with azacitidine induced a CR/CRi rate of 56% as first line treatment in unfit AML patients [115]. Anti‐CD70 cusatuzumab has been shown to eradicate leukemic stem cells in xenotransplantation experiments and to also reduce the frequency of leukemic stem cells in AML patients in combination with azacitidine [116]. Bispecific T‐cell engager such as flotetuzumab (CD3XCD123) or AMG‐330 (CD3XCD33) are more challenging to manage because of the cytokine release syndrome, but may be powerful as single agents especially when the tumor microenvironment is immune‐infiltrated. It has been shown that an immune interferon‐γ signature associated with chemoresistance was predictive of response to flotetuzumab in R/R AML patients, thus suggesting that some immune therapy indications could be guided by a companion biomarker [101]. The use of bispecific antibodies in consolidation or maintenance could also be a valuable option for the eradication of the residual disease, while avoiding the cytokine release syndrome which is correlated with the tumor burden. Many other very promising antibodies are under development in AML [117]. It will be a huge challenge to integrate these new immunotherapies into the extraordinarily changing landscape of AML and adapt them to the disease’ biological heterogeneity.

## 8 NEXT‐GENERATION SEQUENCING TO FOLLOW MINIMAL RESIDUAL DISEASE: WILL WE HAVE OUR CRYSTAL BALL?

Complete remission has been defined morphologically by a threshold of <5% bone marrow blasts together with the recovery of peripheral blood counts and no evidence of extramedullary disease. However, more than 50% of the patients who have reached this stage will ultimately relapse because of a high burden of residual disease that is now better measured, thanks to multiparameter flow cytometry, real‐time quantitative polymerase chain reaction, and more recently by next‐generation sequencing (NGS) [118, 119]. Many studies have shown that the higher the measurable residual disease (MRD), the higher the risk of relapse; and recent guidelines have included MRD in the response criteria [16, 120–124]. Achieving CR with negative MRD is a major goal after first line treatment in order to guide subsequent treatments during consolidation, including allogeneic stem cell transplantation [124]. Furthermore, MRD may become a valuable early surrogate marker of survival endpoints for clinical trials. Indeed, with the introduction of novel drugs in the different phases of post‐remission therapy – such as maintenance and in the treatment of relapse as well as the strong impact of allogeneic stem cell transplantation – it will become very challenging to build successful clinical trials based on OS or event‐free survival endpoints.

The advent of the NGS is critical to the evaluation of MRD and will be more and more used with the advance in NGS technologies which will improve NGS sensitivity and identify very low MRD levels [125]. In addition to the quantitative aspect, this technique will make it possible to detect which mutations persist in remission and those that will drive relapse, leaving room for targeted pre‐emptive therapeutic interventions before morphological relapse, although pre‐leukemic clonal hematopoiesis may interfere with the understanding of the results in some cases. Preliminary results have also shown that single‐cell sequencing could improve the understanding of disease heterogeneity and the dynamics of clonal architecture during morphological remission [126]. Thus, it is likely that maintenance treatment will be soon guided by sequential NGS during the remission phase. In addition, two very important studies have described the pre‐AML mutational landscape that is present in peripheral blood of health individuals several years prior to the diagnosis of overt disease, suggesting that early detection of AML, monitoring and interventional treatment may become a reality in future. [127, 128]

## 9 FUTURE DIRECTIONS

Using new drugs as a single agent or in wise combinations during induction, consolidation and/or maintenance or at relapse, is the major challenge we will have to face in the 10 years to come. In younger patients, the first objective will be to increase the percentage of patients definitely cured by first line treatment. One important point will be to determine if the depth of the response reached by combining intensive chemotherapy and targeted agents eventually followed by novel strategies of immunotherapy will challenge allogeneic stem cell transplantation. This may become a hot topic in the field. In older patients, increasing response rates, duration of response and ultimately OS while maintaining a good quality of life will be major breakthroughs. Like in multiple myeloma, AML in older patients may become a chronic disease with successive lines of treatments, including maintenance, which could be eventually guided by a modern MRD follow‐up. These are only two probable scenarios but others are obviously possible. With this multitude of therapeutic choices and methods to better assess the response and progression of the disease, it is clear that we are coming out of the prehistoric era of AML treatment and that all physicians and biologists involved in AML will be writing an exciting new story.

## CONFLICT OF INTEREST

Research grants from Abbvie, Amgen, Novartis, Celgene, Jazz Pharmaceuticals, Agios, Chugai, MaatPharma, Astellas, Roche, and Daiichi‐Sankyo; an advisory role for Abbvie, Janssen, Jazz Pharmaceuticals, Novartis, Celgene, Otsuka, Astellas, Daiichi‐Sankyo, Macrogenics, Roche, Takeda, and Pfizer.
